# Tumor Size-Dependent Anticancer Efficacy of Chlorin Derivatives for Photodynamic Therapy

**DOI:** 10.3390/ijms19061596

**Published:** 2018-05-29

**Authors:** Ji-Eun Chang, Yang Liu, Tae Heon Lee, Woo Kyoung Lee, Il Yoon, Kwhanmien Kim

**Affiliations:** 1 Department of Thoracic and Cardiovascular Surgery, Seoul National University Bundang Hospital, Seongnam-si, Gyeonggi-do 13620, Korea; iris0515@hanmail.net; 2 Nano Manufacturing Institute, School of Nanoscience and Engineering, Inje University, Gimhae 50834, Korea; gavin881027@163.com (Y.L.); taehun5651@naver.com (T.H.L.); wlee@inje.ac.kr (W.K.L.); 3 Department of Thoracic and Cardiovascular Surgery, Seoul National University College of Medicine, Seoul 03080, Korea

**Keywords:** cancer treatment, chlorin, photodynamic therapy (PDT), photosensitizer, tumor size

## Abstract

Photodynamic therapy (PDT) with a suitable photosensitizer molecule is a promising anticancer treatment. We evaluated two chlorin molecules as potential photosensitizers, methyl pyropheophorbide a (MPPa) and *N*-methoxyl purpurinimide (NMPi), against A549 human lung adenocarcinoma cells in vitro as well as in A549 tumor-bearing mice in vivo. Cell viability, microscopy, and fluorescence-activated cell sorting (FACS) analyses were performed for the in vitro studies. MPPa and NMPi showed high phototoxicity in vitro, which was dependent on the concentration of the photosensitizers as well as the light irradiation time. In the animal study, tumor volume change, tumor surface alterations, and hematoxylin & eosin (H&E) and terminal deoxyribonucleotidyl transferse-mediated dUTP nick-end labelling (TUNEL) staining analyses were performed and compared between small (tumor volume of <50 mm^3^) and large (tumor volume of >50 mm^3^) size of initial tumors. MPPa and NMPi showed high anticancer efficacy against small-size tumors, indicating that early treatment with PDT is effective. Especially, repeated two times PDT with NMPi allowed almost complete eradication against small-size tumors. However, MPPa and NMPi were not effective against large-size tumors. In conclusion, the two chlorin derivatives, MPPa and NMPi, show good anticancer efficacy as promising photosensitizers for PDT in vitro and in vivo. Moreover, their activity in vivo was significantly dependent on the initial tumor size in mice, which confirms the importance of early cancer treatment.

## 1. Introduction

Cancer is one of the most common diseases in the world [1]. Complete tumor eradication is the ultimate goal to achieve perfect healing without recurrence, and recurrence after cancer therapy results in low survival rate as shown in cancer clinical use [2].

Photodynamic therapy (PDT) is a non-invasive and patient-specific cancer therapy using photosensitizers, light, and molecular oxygen in the tumor site for various cancers, such as cervical, endobronchial, esophageal, brain, gastric, and lung cancers [3,4,5,6,7]. PDT possesses several advantages, in that it is tumor selective, minimally invasive, repeatable, and a non-surgical alternative [8,9,10]. The action mechanism of PDT has been introduced elsewhere [5,7]. Upon administration, the photosensitizer molecule selectively accumulates within the tumor tissues. When the photosensitizer is exposed to a specific wavelength of light, it becomes activated from a ground state to an excited state, and, while returning to the ground state, it releases energy that is transferred to oxygen in the body to generate reactive oxygen species (ROS, e.g., singlet oxygen, ^1^O_2_), which induces cellular toxicity to damage cancer cells [11,12].

Chlorins of the porphyrin family are promising second-generation photosensitizer molecules for PDT with a high extinction coefficient at an appropriate wavelength and good ROS photogeneration capabilities [13,14,15]. In addition, chlorins are abundantly available from natural sources such as plants (chlorophyll paste) and algae (*Spirulina maxima*) [16], and can be synthesized in a straightforward manner into chlorin derivatives with various active functional groups on the chlorin moiety [17]. Recently, our group synthesized several chlorin derivatives to evaluate structure–activity relationship [18,19,20,21,22,23]. Among the chlorin derivatives synthesized by our group, we report the activity of two chlorins as photosensitizers, methyl pyropheophorbide a (MPPa) and *N*-methoxyl purpurinimide (NMPi), in this present study [21].

In our previous work, we have shown efficient anticancer activity of PDT using MPPa and NMPi, and their chlorin derivatives against HeLa cells [21], where we focused on the development of long wavelength-absorbing photosensitizers using cationic chlorins. However, 630 nm laser is still common for clinical uses [24,25,26]. Moreover, the commercially available first-generation photosensitizer, Photofrin^®^, produces certain limits to clinical application, such as relatively poor tissue selectivity, low absorption of light, poor tissue penetration of light, skin sensitivity, and adverse effects [26]. Therefore, it is still a great challenge to develop second-generation photosensitizers with high PDT activity as well as without any adverse effects [27]. In this paper, we evaluated two chlorin derivatives, MPPa and NMPi, against A549 cells in vitro as well as in A549 tumor-bearing mice in vivo [28,29,30,31,32].

Furthermore, in this study, we correlated PDT activity with tumor size in mice. Although anticancer efficacy of PDT in various animal models of cancer have been reported, to the best of our knowledge, the effect of PDT according to tumor size has not yet been investigated using tumor animal models. According to clinical reports, there is a remarkable correlation between response rate after PDT and tumor size [15,33,34,35,36]. For early esophageal cancers, it was reported that when the tumor size is up to 1 cm, PDT results in complete regression in 100% patients. However, when the tumor size is 3.1–5.0 cm, only 33.3% of patients achieve complete regression [37]. In addition, one of the recommended criterion for the treatment of lung cancer using PDT is a tumor size less than 1 cm in the greatest diameter [38].

## 2. Results and Discussion

### 2.1. Characterization of Prepared Photosensitizers

**Figure 1:**
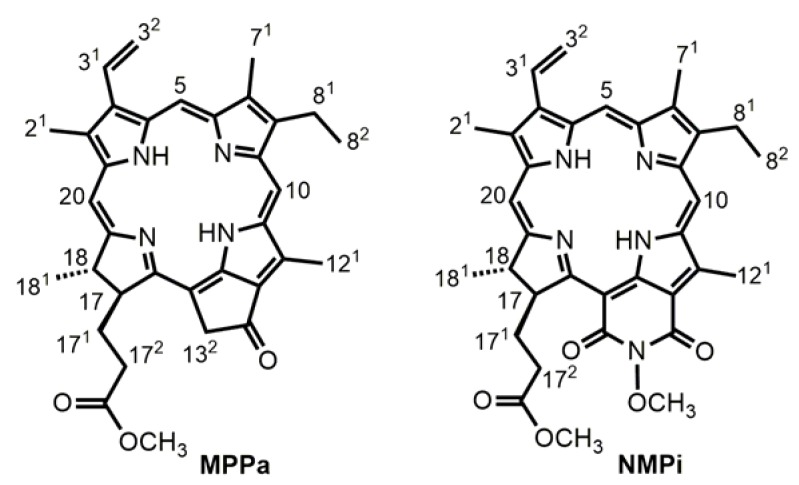
Chemical structures with numbering for methyl pyropheophorbide a (MPPa) and *N*-methoxyl purpurinimide (NMPi).

All characterization data for methyl pyropheophorbide a (MPPa) and *N*-methoxyl purpurinimide (NMPi) (Figure 1) are identical with those in the literature (Figures S1–S3), and are summarized as following [21].

MPPa: ^1^H NMR (CDCl_3_) *δ*: 9.17, 9.06, 8.47 (each s, each 1H, 10-H, 5-H, 20-H), 7.77–7.83 (m, 1H, 3^1^-H), 6.08 (m, 2H, 3^2^-H), 5.20 (d, *J* = 20.0 Hz, 1H, 13^2^-H), 5.05 (d, *J* = 20.0 Hz, 1H, 13^2^-H), 4.40–4.44 (m, 1H, 18-H), 4.20–4.22 (m, 1H, 17-H), 3.42 (q, *J* = 7.3 Hz, 2H, 8^1^-CH_2_), 3.01, 3.31, 3.50, 3.61 (each s, 12H, OCH_3_ + CH_3_), 2.51–2.53 (m, 2H, 17^2^-CH_2_), 2.21–2.27 (m, 2H 17^1^-CH_2_), 1.80 (d, *J* = 7.4 Hz, 3H, 18^1^-CH_3_), 1.56 (t, *J* = 7.5 Hz, 3H, 8^2^-CH_3_), 0.75 (br, 1H, NH), −1.92 (br, 1H, NH); UV-Vis (nm) (log *ε*): 412.90 (1.54), 509.90 (1.07), 539.80 (1.01), 610.70 (0.93), 667.90 (1.49); Anal. calcd for C_34_H_36_N_4_O_3_ C 74.43; H 6.61; N 10.21. Found C 74.38, H 7.14, N 10.06.

NMPi: ^1^H NMR (CDCl_3_) *δ*: 8.53, 9.33, 9.58 (each s, each 1H, 10-H, 5-H, 20-H), 7.86 (dd, *J* = 17.8 Hz, 11.6 Hz, 1H, 3^1^-H), 6.29 (d, *J* = 17.8 Hz, 1H, 3^2^-H), 6.17 (d, *J* = 11.6 Hz, 1H, 3^2^-H), 5.28 (d, *J* = 9.7 Hz, 1H, 17-H), 4.35 (q, *J* = 7.4 Hz, 1H, 18-H), 4.39 (s, 3H, NOCH_3_), 3.16, 3.33, 3.58, 3.67 (each s, each 3H, OCH_3_ + CH_3_), 3.67 (q, *J* = 7.6 Hz, 2H, 8^1^-CH_2_), 1.90–2.08, 2.40–2.56, 2.73–2.85 (each m, 4H, 17^1^ + 17^2^-CH_2_), 1.72 (d, *J* = 7.3 Hz, 3H, 18^1^-CH_3_), 1.63 (t, *J* = 7.6 Hz, 3H, 8^2^-CH_3_), 0.14, 0.23 (each br s, each 1H, 2 × NH); UV-Vis: (nm) (log *ε*): 410.00 (1.51), 481.10 (0.84), 551.50 (1.28), 707.10 (1.47); Anal. calcd for C_35_H_37_N_5_O_5_(CH_3_OH) C 67.59; H 6.46; N 10.95. Found C 67.95, H 6.98, N 10.95.

### 2.2. In Vitro Phototoxicity

The photoactivity and dark toxicity of MPPa and NMPi were evaluated against A549 cells by MTT assay at various concentrations (1–20 µM) after 3 and 24 h incubation times (Tables S1 and S2). For the photoactivity study, A549 cells were irradiated to give total light dose of 2 J/cm^2^ for 15 min, which is the same experimental condition as our previous cell viability study [21].

**Figure 2:**
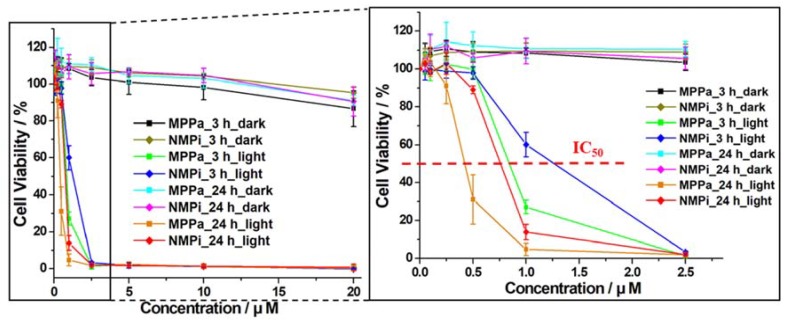
In vitro phototoxicity (total light dose 2 J/cm^2^ for 15 min) and dark toxicity against A549 cells by 3-(4,5-Dimethylthiazol-2-yl)-2,5-diphenyltetrazolium bromide) (MTT) assay after 3 and 24 h incubation times at various concentrations (1–20 µM) of MPPa and NMPi (left), and expanded figure for low concentrations (right). Error bars represent the SD of three replicate experiments. Red dashed line at 50% cell viability presents IC_50_ value (half maximal inhibitory concentration).

We compared photoactivities of MPPa and NMPi against A549 and HeLa cells. Figure 2 exhibited negligible dark toxicity until 20 µM, while upon photoirradiation, the cell viability decreased for MPPa and NMPi consistent with the increase in their concentrations as well as incubation time. Photoactivity of IC_50_ values of MPPa and NMPi against A549 and HeLa cells are summarized in Table 1. MPPa showed higher photoactivity than NMPi against both A549 and HeLa cells. It is interesting that MPPa presented higher photoactivity against HeLa cells than A549 cells, while NMPi had higher photoactivity against A549 cells than HeLa cells. This may be because the two cell lines have different morphology, histology, and biochemistry based on differences in phenotypic and genotypic characteristics and origins determining resistance and biological response [29,30].

**Figure 3:**
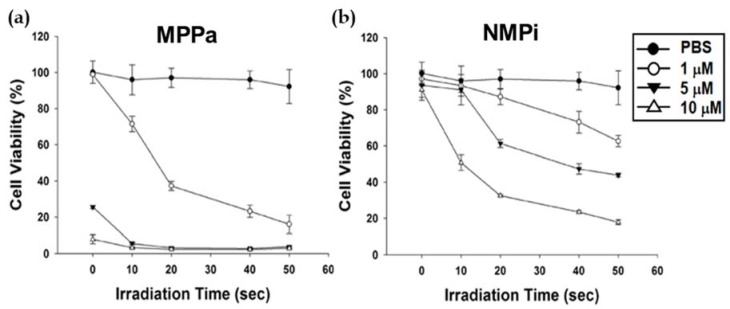
In vitro phototoxicity (630 nm laser, 400 mW/cm^2^) and dark toxicity against A549 cells by CCK-8 at 4 h incubation time after treating with PBS or various concentrations (1, 5, and 10 µM) of (**a**) MPPa or (**b**) NMPi followed by light irradiation for 0, 10, 20, 40, or 50 s (0, 4, 8, 16, or 20 J/cm^2^, respectively). Error bars represent the SD of three replicate experiments.

Before starting the in vivo study, we confirmed the suitable experimental condition of the in vivo system with a 630 nm laser (400 mW/cm^2^) as well as confirmed the drug dose of the two chlorins, MPPa and NMPi. Therefore, we reevaluated light and drug doses against A549 cells in vitro. Figure 3 shows in vitro cell viabilities against A549 cells after 4 h incubation time by CCK-8 with PBS (control), MPPa, and NMPi at a concentration range of 1–10 µM. Dark toxicity of NMPi was negligible until 10 µM. MPPa presented high dark toxicity at 5 µM and 10 µM concentrations, which was not consistent with that obtained by MTT assay shown in Figure 1 (negligible dark toxicity until 20 µM). In the previous study, MPPa and NMPi had no dark toxicity against HeLa cells until 10 µM [21].

Phototoxicity of MPPa and NMPi was evaluated with the photoirradiation time set at 10–50 s (4–20 J/cm^2^). Under laser treatment, photoactivity of PBS was negligible. However, MPPa and NMPi exhibited significantly high photoactivity that were dependent on their concentrations as well as the irradiation time. MPPa showed better phototoxicity compared with NMPi. Furthermore, MPPa and NMPi showed better photoactivity than chlorin e6-loaded chitosan nanoparticles reported by Wang et al. [31].

The results of in vitro cell viability studies (Figure 2 and Figure 3) clearly suggest that effective intracellular uptake of MPPa and NMPi by A549 cells follows photodynamic activity to induce apoptosis in tumor cells.

**Figure 4:**
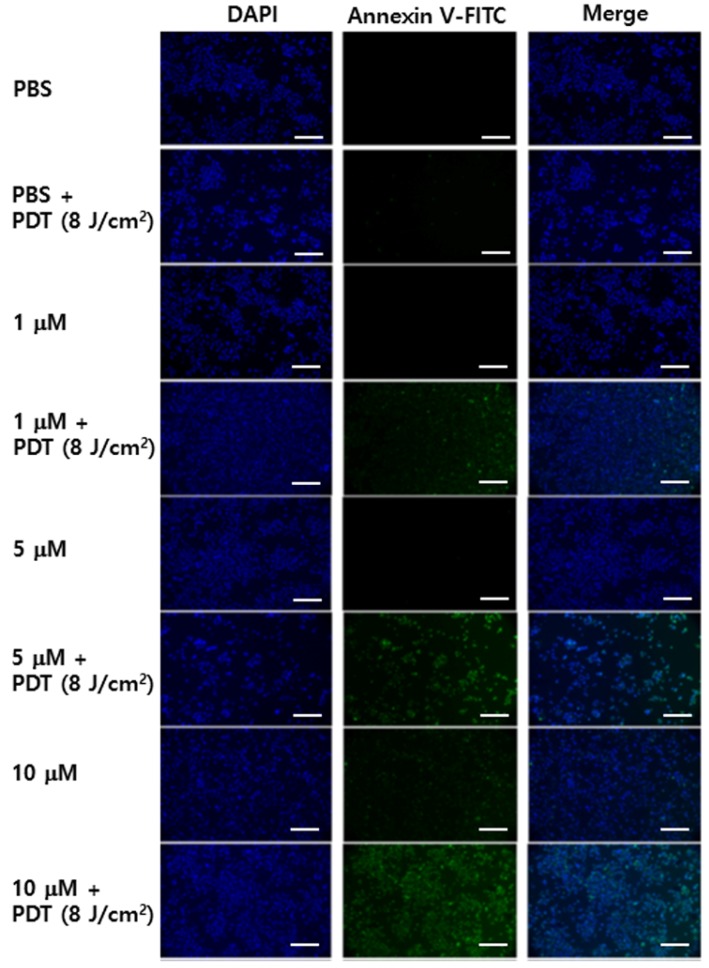
Microscopic analysis of apoptotic A549 cells by annexin V-FITC staining after treating PBS or various concentrations (1–10 µM) of NMPi followed by light irradiation for 0 or 8 J/cm^2^. DAPI stain (blue, first column) and annexin V-FITC stain (green, second column) indicate nuclei and apoptotic cells, respectively. Bars, 100 µm.

Microscopic analysis (Figure 4) was performed to visualize apoptotic cell death caused by PDT against A549 cells by annexin V-FITC staining after treating with PBS or various concentrations (1–10 µM) of NMPi followed by light irradiation for 0 or 8 J/cm^2^. Nuclei and apoptotic cells were stained with DAPI (blue color, first column) and annexin V-FITC (green color, second column). PDT with NMPi along with irradiation of 8 J/cm^2^ revealed increased effect of PDT corresponding to concentration, which was consistent with the results of in vitro phototoxicity in Figure 1 and Figure 2. However, PBS and PS only without PDT showed no apoptotic cells, except at 10 µM NMPi concentration, which exhibited some apoptotic cells due to the low dark toxicity.

**Figure 5:**
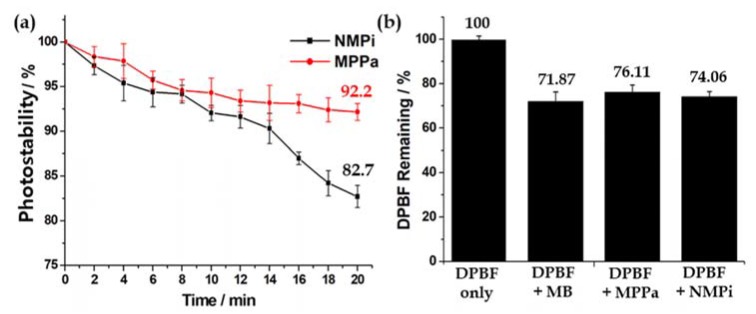
(**a**) Photostability (photobleaching) of MPPa and NMPi in DMSO (4 µM) with light irradiation up to 20 min (total light energy 2.7 J/cm^2^), measured by UV-Vis absorption. (**b**) 1,3-Diphenylisobenzofuran (DPBF) (50 µM in DMSO) remaining (%) at 418 nm after photoirradiation (total light dose 2 J/cm^2^ for 15 min) in the absence (control, DPBF only) and presence of 1 µM MB, MPPa, and NMPi. Error bars represent the SD of three replicate experiments.

We tested photostability (photobleaching) of MPPa and NMPi in DMSO (4 µM) with light irradiation up to 20 min (total light energy 2.7 J/cm^2^) as shown in Figure 5a (Figures S3 and S4) [12]. MPPa (92.2%) showed better photostability than NMPi (82.7%), which may be why MPPa has better photoactivity than NMPi.

To quantify the relative photoactivity of MPPa and NMPi in the absence of tumor cells, ^1^O_2_ photogeneration was measured by 1,3-diphenylisobenzofuran (DPBF) as a selective ^1^O_2_ acceptor (Figure 5b). NMPi presented slightly better ^1^O_2_ photogeneration than MPPa, and NMPi was almost comparable to methylene blue (MB) as a standard ^1^O_2_ sensitizer [39]. This result was consistent with the cell viability results shown in Figure 2 and Figure 3.

**Figure 6:**
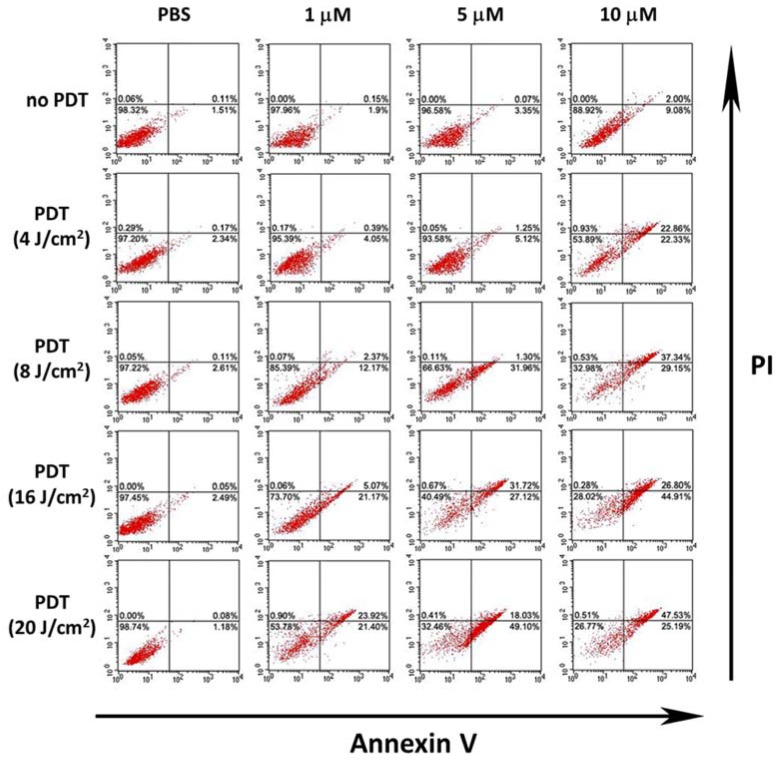
FACS analysis of A549 cells after treating with PBS or various concentrations (1, 5, and 10 µM) of NMPi followed by light irradiation at 0, 4, 8, 16, or 20 J/cm^2^. The cells were categorized as follows: both annexin V-FITC and PI negative cells, undamaged; annexin V-FITC positive and PI negative cells, early apoptotic; both annexin V-FITC and PI positive cells, late apoptotic; and annexin V-FITC negative and PI positive cells, either late apoptotic or necrotic.

To calculate quantitative cell death by either apoptosis or necrosis, fluorescence-activated cell sorting (FACS) analysis was performed (Figure 6) against A549 cells by double staining with annexin V-FITC and PI. Positive staining by annexin V-FITC indicates early apoptosis. Both staining by annexin V-FITC and PI show late apoptosis. Positive staining by PI indicates necrosis. Total amount of apoptotic cells (sum of early and late apoptosis) increased with an increase in irradiation energy from 4 to 20 J/cm^2^ PDT (45.19%, 66.49%, 71.71%, and 72.72%), which was consistent with the in vitro phototoxicity results in Figure 1 and Figure 2 as well as the microscopic analysis in Figure 4.

### 2.3. In Vivo Photodynamic Therapeutic Efficacy

In the in vitro result, CCK-8 assay showed some dark toxicity in MPPa (Figure 3), however, MTT assay revealed there was almost no dark toxicity for both compounds, MPPa and NMPi until 20 µM concentration (Figure 2). PBS was used as a representative control group, because the drug only or light alone may give negligible activity result in the animal study due to no PDT action like in PBS alone (absence of the drug and light). We used 630 nm laser with output power of 400 mW/cm^2^, which was the same experimental condition as that in our previous study; this condition allowed suitable irradiation with enough power to afford phototoxicity but no damage to cells or tissues by itself without photosensitizer administration. During the animal study, there were no side effects, which indicates that the drug alone or light alone could not make any activity results as well as the in vivo experimental conditions made no damage except the PDT action (the absence of another controls).

Therapeutic efficiency of the two photosensitizers, MPPa and NMPi, in A549 tumor-bearing mice was evaluated. Double drug injections were performed on Days 0 and 2, followed by laser irradiations (200 J/cm^2^) one day after each injection on Days 1 and 3. In vivo tumor volume in A549 tumor-bearing mice (small-tumor groups (<50 mm^3^) and large-tumor groups (>50 mm^3^)) were measured and compared based on different photosensitizers and tumor sizes.

**Figure 7:**
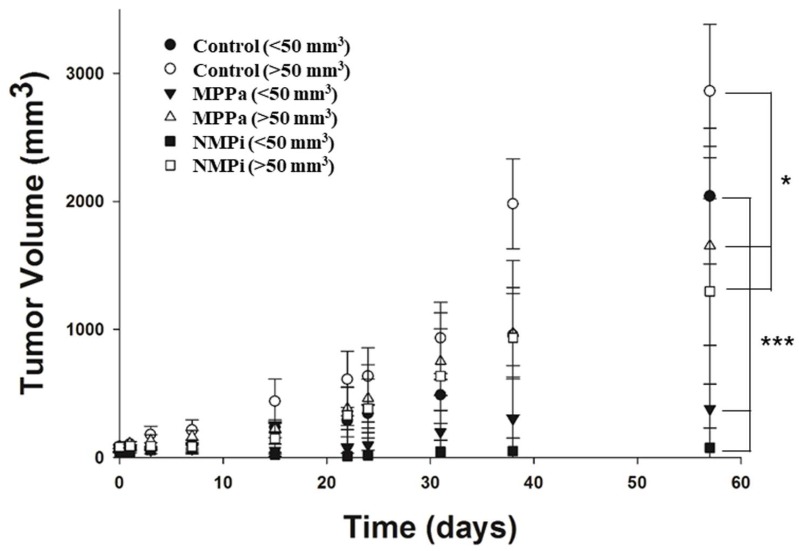
In vivo tumor volume changes in A549 tumor-bearing mice (small-tumor groups <50 mm^3^, and large-tumor groups >50 mm^3^) after double injections (on Days 0 and 2, repeated two times PDT) of PBS, MPPa (2 mg/kg), or NMPi (2 mg/kg) followed by light irradiation (200 J/cm^2^) one day after each injection (on Days 1 and 3, repeated two times PDT). Data are presented as means ± SD (*n* = 5). The tumor volume (mm^3^) was calculated as (length × width^2^)/2. Therapeutic effect was higher in the order of NMPi (<50 mm^3^) > MPPa (<50 mm^3^) > NMPi (>50 mm^3^) > MPPa (>50 mm^3^). *** *p* < 0.001 and * *p* < 0.05 (one-way ANOVA) indicate values that differ significantly from control (PBS) group in small-tumor groups (<50 mm^3^) and in large-tumor groups (>50 mm^3^), respectively.

Data are presented as means ± SD (*n* = 5) in Figure 7. As expected, therapeutic effect was higher in the order of: NMPi (<50 mm^3^) > MPPa (<50 mm^3^) > NMPi (>50 mm^3^) > MPPa (>50 mm^3^). It is noted that NMPi (<50 mm^3^) caused almost complete tumor eradication, and MPPa (<50 mm^3^) significantly reduced tumor volume under PDT. This result showed that under in vivo conditions, NMPi and MPPa subgroups showed so much higher therapeutic effect and a significantly lower tumor volume than control (PBS) in A549 tumor-bearing mice in both small- (*p* < 0.001, one-way ANOVA) and large-size (*p* < 0.05, one-way ANOVA) tumor groups. This result confirms the importance of PDT effect with suitable photosensitizer as promising anticancer treatment. However, therapeutic effects of MPPa and NMPi on mice with large-tumors (>50 mm^3^) were not high. These results indicate that initial tumor size strongly affects therapeutic effect under PDT [15,33].

Interestingly, NMPi showed better PDT anticancer efficacy than MPPa in vivo (Figure 7). However, there is no significant (*p* > 0.05, one-way ANOVA) difference between NMPi and MPPa subgroups in both small- and large-size tumor groups. The increase in apoptotic cells induced by the photodynamic action supports that the anticancer effect of PDT strongly depends on the concentration of NMPi (Figure 2 and Figure 3) and the total energy of light irradiation (Figure 4 and Figure 6).

Moreover, in the in vivo study, the therapeutic effect of NMPi at lower drug and light doses (drug dose of 2 mg/kg and total light dose of 200 J/cm^2^) was comparable to that of the chlorin e6-loaded hyaluronic acid nanoparticles reported by Cai et al. (660 nm laser with 160 mW/cm^2^ for 30 min (total light dose, 288 J/cm^2^); drug dose, 5 mg/kg; initial tumor size, 50–100 mm^3^) [32]. This result suggests that the treatment is effective against small-size tumors, while large-size tumors need higher drug and light doses to allow complete tumor eradication.

Tumor volume changes shown in Figure 7 clearly indicate that early treatment (small-size initial tumors) is important to obtain remarkable therapeutic effect [34,35,36]. Therefore, late treatment (large-size initial tumors) may be effective in PDT only with higher drug and light doses. NMPi for small-size initial tumors (<50 mm^3^) revealed excellent PDT activity with almost complete tumor eradication under the treatment condition.

**Figure 8:**
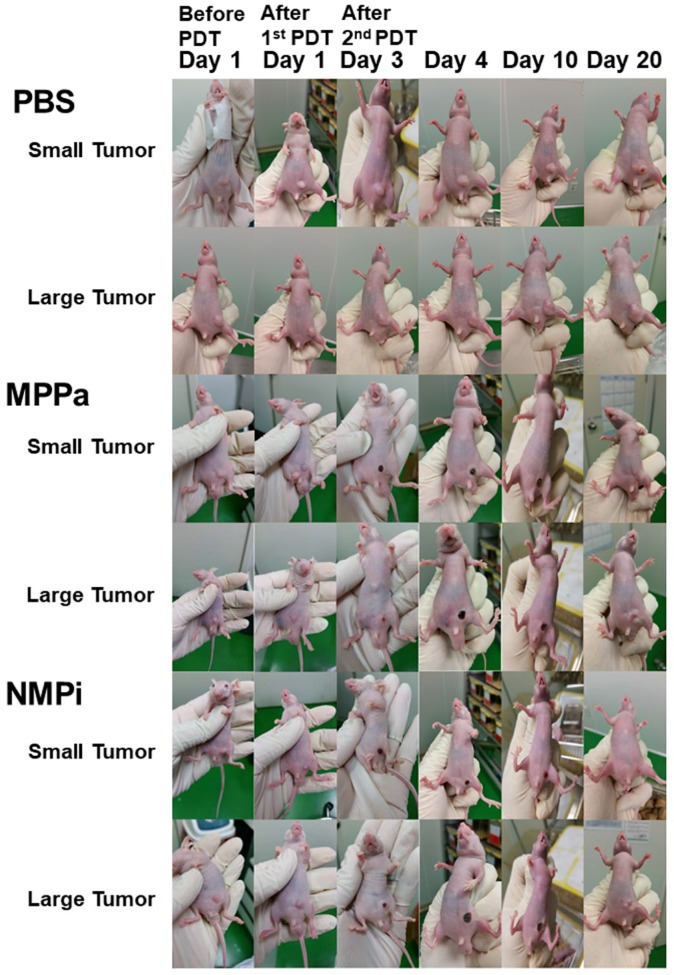
Tumor surface alterations of A549 tumor-bearing mice (small-tumor group <50 mm^3^ and large-tumor group >50 mm^3^) after double intravenous injection (on Days 0 and 2, repeated two times PDT) of PBS, MPPa (2 mg/kg), and NMPi (2 mg/kg) with 200 J/cm^2^ light irradiation on one day after each injection (on Days 1 and 3, repeated two times PDT). Mice in the small-tumor group (<50 mm^3^) treated with MPPa and NMPi under PDT revealed complete tumor eradication on Day 20. However, in large-tumor group (>50 mm^3^), tumor tissues were not completely eliminated.

The images for tumor surface alterations of A549 tumor-bearing mice (small- and large-tumor groups) after double intravenous injections (on Days 0 and 2, repeated two times PDT) of PBS, MPPa, and NMPi with 200 J/cm^2^ light irradiation one day after each injection (on Days 1 and 3, repeated two times PDT) are shown in Figure 8. On the day of second PDT (on Day 3), mice in MPPa and NMPi groups showed hemorrhage where the light was delivered, and the tumor tissues were severely damaged due to the photogeneration of ROS followed by direct cellular damage and destruction of tumor tissue [3,4,5,6,7]. Mice in the small-tumor group (<50 mm^3^) treated with MPPa and NMPi under PDT had complete tumor eradication on Day 20. However, in large-tumor group (>50 mm^3^), tumor tissues were not completely eliminated.

In addition, the images of tumor surface alteration shown in Figure 8 indicate the reaction to light irradiation. On Day 4, one day after the second PDT, immediate reaction with damaged apoptotic tissues (dark color) in both small- and large-tumor groups was observed. The damaged tissues were healed on Day 20, 17 days after the second PDT, and small tumors treated with MPPa and NMPi completely disappeared; however, large tumors expressed regrowth.

PDT has a good advantage over other cancer treatments, in that it is a repeatable treatment. In this research, we also used repeatable PDT, such as two times of PDT on Day 1 and 3. We believe that repeatable PDT is important to allow complete tumor healing in the small-tumor groups. Wu et al. used repeated PDT for four times and reported high antibacterial efficacy [40]. Na et al. developed a repeatable endoscopic PDT-stent [41].

In this study, our in vivo results were better than our previous in vitro and in vivo results with use of the same drug (2 mg/kg) and light (200 J/cm^2^ by 630 nm with 400 mW/cm^2^ for 500 s) doses using hematoporphyrin-modified doxorubicin-loaded nanoparticles [42] as well as hypocrellin B and paclitaxel loaded nanoparticles [43], not only because of the repeated two times PDT, but also because of the early treatment when the tumor size was small.

This result has a good agreement with previous clinical trials results. Five-year survival rate (%) is highly affected by tumor sizes, such as 92.0% for ≤110 cm^3^ and 62.2% for >110 cm^3^ [34]. William Jr. et al. have shown good relationship between tumor size and survival in non-small-cell lung cancer in their study after tumor sizes were categorized into four subgroups: ≤2 cm, 2–5 cm, 5–7 cm, and >7 cm [35]. Patients with a tumor size ≤2 cm have longer survival as reported by Rami-Porta et al. [36].

**Figure 9:**
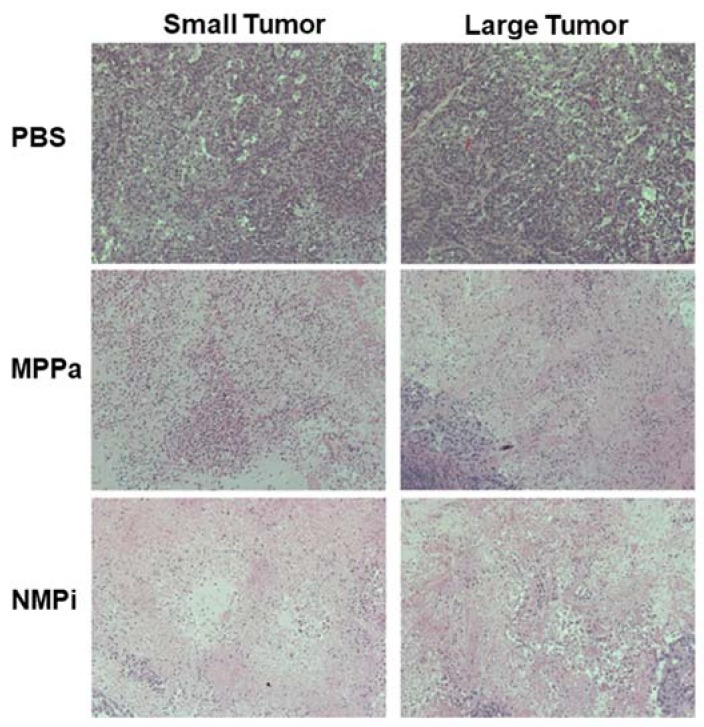
Hematoxylin and eosin (H&E) staining of tumor tissues in A549 tumor-bearing mice (small-tumor group <50 mm^3^ and large-tumor group >50 mm^3^) on Day 15, after double intravenous injection (on Days 0 and 2, repeated two times PDT) of PBS, MPPa (2 mg/kg) and NMPi (2 mg/kg) with 200 J/cm^2^ light irradiation on one day after each injection (on Days 1 and 3, repeated two times PDT). Mice in the small tumor group treated with NMPi presented the most complete tumor cell eradication, which was also consistent with the result of tumor surface alterations in Figure 8. The magnification is 40×.

Hematoxylin and eosin (H&E) staining images of tumor tissues in A549 tumor-bearing mice (small- and large-tumor groups) on Day 15, after double intravenous injection (on Days 0 and 2, repeated two times PDT) of PBS, MPPa, and NMPi with 200 J/cm^2^ light irradiation on one day after each injection (on Days 1 and 3, repeated two times PDT) are shown in Figure 9.

**Figure 10:**
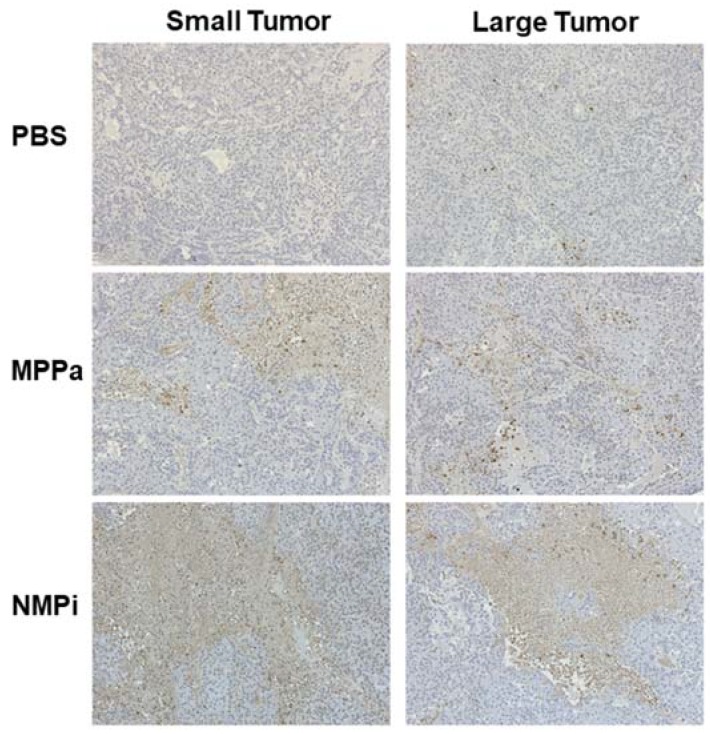
Terminal deoxyribonucleotidyl transferse-mediated dUTP nick-end labelling (TUNEL) staining of tumor tissues in A549 tumor-bearing mice (small-tumor group <50 mm^3^ and large-tumor group >50 mm^3^) on Day 15, after double intravenous injection (on Days 0 and 2, repeated two times PDT) of PBS, MPPa (2 mg/kg), and NMPi (2 mg/kg) with 200 J/cm^2^ light irradiation on one day after each injection (on Days 1 and 3, repeated two times PDT). This result also had a good agreement with therapeutic effects observed in the H&E staining images in Figure 8. The magnification is 40×.

The tumor cells were damaged in the same order as for the tumor size reduction in Figure 7. Mice in the small-tumor group treated with NMPi presented the most complete tumor cell eradication, which is also consistent with the result of tumor surface alterations in Figure 8. Furthermore, in Figure 10, we show the results of terminal deoxyribonucleotidyl transferse-mediated dUTP nick-end labelling (TUNEL) staining for detecting apoptotic DNA fragmentation in A549 tumor-bearing mice on Day 15. This result also had a good agreement with therapeutic effects observed in the H&E staining images in Figure 9.

Consequently, under the PDT, small-tumor size groups have shown better photodynamic activity result than large-tumor size groups, and treatment with NMPi showed higher therapeutic effect than treated with MPPa.

## 3. Materials and Methods

### 3.1. Materials

RPMI-1640 medium, penicillin-streptomycin, and fetal bovine serum (FBS) were obtained from Gibco Life Technologies, Inc. (Grand Island, NY, USA). Phosphate buffered saline (PBS) was obtained from Sigma-Aldrich (St. Louis, MO, USA). 3-(4,5-Dimethylthiazol-2-yl)-2,5-diphenyltetrazolium bromide) MTT kit (EZ-CYTOX, DOGEN, Seoul, Korea), was obtained from DOGEN Bio.

### 3.2. Synthesis of Photosensitizers

**Scheme 1:**
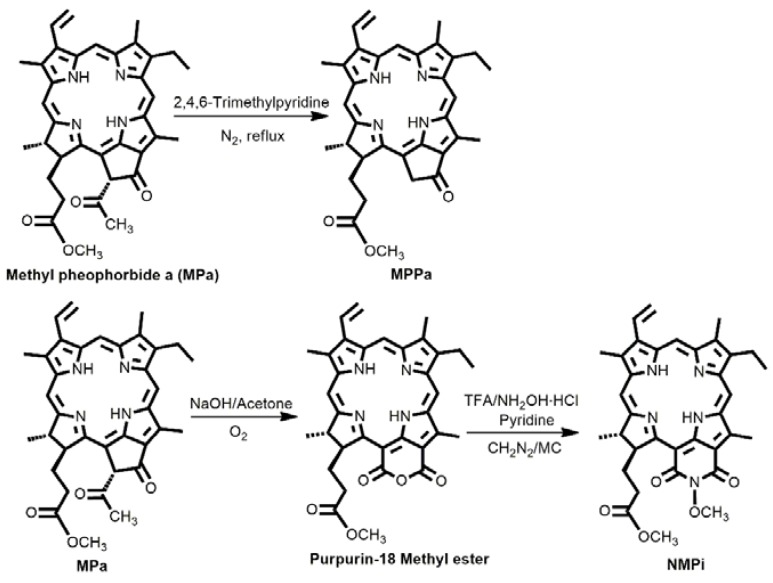
Synthetic procedure of MPPa and NMPi.

Chlorophyll a-based photosensitizers, MPPa and NMPi, were synthesized according to a modified literature procedure by our group (Scheme 1) [21].

MPPa: Methyl pheophorbide a (MPa) was obtained from an extraction method of chlorophyll paste with 5% acidic methanol followed by column separation (SiO_2_ and eluent 2% acetone/CH_2_Cl_2_). Then, MPa (500 mg) was dissolved in 100 mL 2,4,6-trimethylpyridine under stirring at reflux in a nitrogen environment. After 5 h, 2,4,6-trimethylpyridine was removed by evaporation under high vacuum, and the residue was purified by column separation (SiO_2_ and eluent of 2% MeOH/CH_2_Cl_2_) to give a dark green solid (yield 85%).

NMPi: Purpurin-18 methyl ester was obtained by reacting MPa with basic acetone in oxygen followed by column separation (SiO_2_ and eluent 2% acetone/CH_2_Cl_2_). Purpurin-18 methyl ester (200 mg) was reacted with hydroxylamine hydrochloride (500 mg, excess) in pyridine under stirring at room temperature for 5 h. After that, the reaction solvent was removed by washing with aqueous HCl (2 M, 3 ×100 mL) and water (3 × 200 mL). After removing all the solvent, the residue was dissolved in CH_2_Cl_2_ (10 mL) and reacted with 5 mL diazomethane for 5 min. The residue was purified by column separation (SiO_2_ and eluent of 3% MeOH/CH_2_Cl_2_) to give a dark purple solid (yield 75%).

### 3.3. In Vitro Phototoxicity

#### 3.3.1. MTT Assay According to Concentration of Photosensitizer

A549 cells were placed in a 96-well plate at 1 × 10^4^ cells/well (100 µL) to prepare an adherent culture. After 24 h, the medium was removed, the cells were washed twice with PBS, and MPPa and NMPi at various concentrations (0.1–20 µM in 100 μL of the medium) were added to each well. After 24 h, the solutions were discarded, and the cells were washed thrice with PBS, followed by the addition of fresh medium (100 μL/well). The cells were then irradiated with a BioSpec LED (610–710 nm, 2 J/cm^2^) from a distance of 20 cm for 15 min, followed by an MTT assay to evaluate their response to PDT. In the assay, MTT solution (10 μL) was added to each well followed by culturing in an incubator for 1 h. Subsequently, the absorbance at 450 nm was measured with an ELISA-reader at 3 and 24 h after photoirradiation. All assays were performed in triplicate in three independent experiments.

#### 3.3.2. CCK-8 Assay According to Irradiation Time and Concentration of Photosensitizer

A549 (human lung adenocarcinoma, ATCC, Manassas, VA, USA) cells were seeded in 24-well cell culture plates (Nunc, Roskilde, Denmark) at a density of 1 × 10^5^ cells/well in RPMI-1640 medium containing 10% (*v*/*v*) FBS and 1% (*w*/*v*) penicillin–streptomycin and cultured for 24 h at 37 °C with 5% CO_2_ and 95% air atmosphere in a humidified incubator. After 24 h, the cells were washed with PBS and treated with PBS (control), or various concentrations (1, 5, and 10 µM) of MPPa or NMPi. Both compounds were dissolved in DMSO (1 mM, 1 mL) and were diluted with PBS, and then 1 mL of the solutions were added to each well. After 4 h of incubation, the cells were washed twice with PBS, and then fresh RPMI-1640 medium was added.

The tumor cell viability assay was performed using a Cell Counting Kit-8 (CCK-8) (Dojindo Molecular Technologies, Gaithersburg, MD, USA) [44]. The drug-treated cells were irradiated with a PDT laser (Diomed Inc., Andover, MA, USA) at 630 nm and 400 mW/cm^2^ for various periods of time (0, 10, 20, 40, and 50 s corresponding to 0, 4, 8, 16, and 20 J/cm^2^, respectively) and incubated for 24 h in the dark. The cells were washed twice with cold PBS and 10 μL of CCK-8 solution and 100 μL of culture medium were added to each well. Then, the cells were incubated in the dark for an additional 2 h. The absorbance was read at a wavelength of 450 nm with a microplate reader (Spectramax plus 384, Molecular Devices Corporation, Sunnyvale, CA, USA). All assays were performed in triplicate in three independent experiments.

### 3.4. Microscopic Analysis

The cells were irradiated with a PDT laser (630 nm, 400 mW/cm^2^) for 0 and 20 s (0 and 8 J/cm^2^, respectively), incubated in the dark for 24 h, and washed twice with cold PBS. The annexin V-FITC fluorescence microscopy kit (BD bioscience, San Jose, CA, USA) was used to identify apoptotic cells [45]. The cells were stained with both annexin V-FITC and DAPI (Vector Laboratories, Burlingame, CA, USA), and the apoptotic cells were counted on the fluorescence microscope (Axioskop 40, Carl Zeiss, Göttingen, Germany).

### 3.5. Singlet Oxygen Photogeneration Study

**Figure 11:**
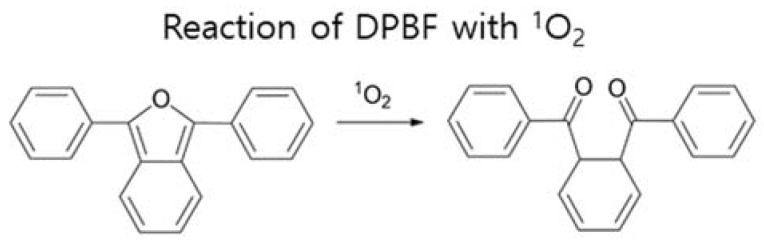
Reaction of DPBF with ^1^O_2_ to decrease its absorbance at 418 nm, which depends on the amount of ^1^O_2_ photogeneration.

DPBF study was carried out to evaluate relative photogeneration of singlet oxygen (^1^O_2_) after photoirradiation (total light dose 2 J/cm^2^) [46]. After DPBF bind with ^1^O_2_, the furan ring on DPBF opens up to generate a diketone, resulting in the decay of absorbance of DPBF at 418 nm. DPBF was used as a selective ^1^O_2_ acceptor, being bleached upon reaction with ^1^O_2_. Four sample solutions of DPBF in DMSO (50 µM) containing DPBF only (50 µM, control sample), DPBF + methylene blue (MB) (1 µM), DPBF + MPPa (1 µM), and DPBF + NMPi (1 µM) were prepared in the dark. All samples were placed in a 96-well plate and covered with aluminum foil. The samples were irradiated (2 J/cm^2^) for 15 min. After irradiation, visible spectra of the sample solutions were recorded, and the normalized absorbances of DPBF at 418 nm were reported (Figure 11). The ^1^O_2_ photogeneration activities of MPPa and NMPi were compared based on the different absorbance decay profiles of each sample relative to that of the DPBF control sample.

### 3.6. FACS Analysis

The cells were irradiated with a PDT laser (630 nm, 400 mW/cm^2^) for 0, 10, 20, 40, and 50 s (0, 4, 8, 16, and 20 J/cm^2^, respectively). The irradiated cells were incubated in the dark for 24 h and washed twice with cold PBS. The cells were stained with both annexin V-FITC and PI (BD Biosciences, San Jose, CA, USA) [47]. The stained cells were resuspended in 1 mL of 1× binding buffer and then 100 μL of the sample solution was transferred to a 5 mL round-bottom tube. Annexin V-FITC (5 μL) and PI (5 μL) were added into the sample solution and incubated in the dark for 15 min at room temperature. Finally, binding buffer (400 μL) was added and the apoptotic cells were detected by flow cytometry (FACSCalibur, BD Biosciences) using CellQuest software (BD Immunocytometry Systems, Mountain View, CA, USA). The excitation wavelengths of PI and annexin V-FITC were 488 nm and 635 nm, respectively. The emission wavelengths of PI and annexin V-FITC were 610 ± 20 nm and 660 ± 20 nm, respectively. Acquired cells on the flow cytometer were collected per 10,000 events.

### 3.7. Animals and Tumor Model

BALB/c male nude mice (6–7 weeks, 20–22 g) were purchased from Orientbio (Gyeonggi-do, Korea). All animal study protocols were approved by the Institutional Animal Care and Use Committee of Seoul National University Bundang Hospital on 20 December 2016 (BA1612-214/081-01). A549 cells at 1 × 10^6^ in 0.1 mL RPMI-1640 medium were injected into the left flanks of all mice subcutaneously at the same time [32]. During treatment, mice were anesthetized with CO_2_ at a flow rate of 0.1 L/min. The tumor size of each mouse was measured by a caliper for 57 days to investigate PDT efficacy. The tumor sizes (mm^3^) were calculated as the volume (tumor length) × (tumor width)^2^/2 [48]. Tumor volumes of A549 tumor-bearing mice reached <50 mm^3^ (small-size) and >50 mm^3^ (large-size) after seven and ten days, respectively.

### 3.8. In Vivo Anticancer Efficacy in Tumor-Bearing Mice

In vivo anticancer efficacy was performed using A549 tumor-bearing mice. The mice were divided into two groups according to the tumor size; a small-tumor group (<50 mm^3^) and a large-tumor group (>50 mm^3^). Then, the two groups were classified into three subgroups: control, MPPa and NMPi (*n* = 5 each). The mice were injected twice (on Days 0 and 2, repeated PDT) with PBS, MPPa (2 mg/kg), or NMPi (2 mg/kg) via the tail vein. Twenty-four hours after each injection, tumors were irradiated by a PDT laser (630 nm with 400 mW/cm^2^) for 500 s (total light dose of 200 J/cm^2^) twice (on Days 1 and 3, repeated PDT). The surface changes at the tumor site were also monitored.

### 3.9. Histology Examination

For histology analysis, the mice (one mouse in each group) were sacrificed on Day 15, and the tumor tissues of mice in each subgroup from the small- and large-tumor groups were excised, fixed in 10% formalin, and then embedded in paraffin. Tissue sections were deparaffinized in xylene, dehydrated in graded alcohols, and washed in distilled water. The tissues were sectioned into 5-μm-thick slices for H&E staining.

### 3.10. TUNEL Assay for Apoptosis

For TUNEL assay, the mice (one mouse in each group) were sacrificed on Day 15, and the tumor tissues of mice in each subgroup from the small- and large-tumor groups were excised. Apoptotic cell death in paraffin-embedded tumor tissue sections was detected using the in situ Cell Death Detencion Kit according to the manufacturer’s method.

### 3.11. Statistical Analysis

All data are presented as means ± standard deviation (SD). The statistical significance among the three groups (control, MPPa and NMPi) was determined by one-way analysis of variance (ANOVA) and multiple comparisons (Fisher’s method as a post-hoc test) with the Statistical Program for Social Sciences software (SPSS). In all analyses, *p* < 0.05 was considered statistically significant difference.

## 4. Conclusions

In this study, we demonstrated that the two chlorin derivatives as second-generation photosensitizers, MPPa and NMPi, show high anticancer efficacy by PDT against A549 human lung adenocarcinoma cells in vitro as well as in A549 tumor-bearing mice in vivo. Tumor cell viability was evaluated by both MTT and CCK-8 assays, and we found that PDT activity significantly depends on the irradiation time and the concentration of the photosensitizer. MPPa showed better phototoxicity than NMPi. For the in vivo study, we used two times repeatable PDT method and compared the results between the small-size (≤50 mm^3^) and the large-size (>50 mm^3^) tumor groups. As expected, mice in the small-tumor groups healed better than those in the large-tumor groups.

Importantly, in our in vivo system, both MPPa and NMPi decreased tumor growth than the control. More importantly, NMPi has shown excellent tumor eradication not only because of the repeatable two times PDT as an advantage of PDT compared with other anticancer treatments, but also because of the small-size of tumor correlating with early cancer treatment.

To the best of our knowledge, this study is the first paper to show relative anticancer results by PDT corresponding to the different tumor sizes. Therefore, these results could be useful to develop new potential photosensitizers for PDT as well as for understanding the relationship between tumor size and anticancer efficacy.

## Figures and Tables

**Table 1:** IC_50_ values of MPPa and NMPi against A549 ^a^ and HeLa ^b^ cells at 3 h and 24 h, and 12 h incubation times after irradiation (total light dose 2 J/cm^2^ for 15 min), respectively.

Cell Line (Assay)	Incubation Time (h)	MPPa (µM)	NMPi (µM)
A549 ^a^ (MTT)	3	0.89	1.15
	24	0.44	0.73
HeLa ^b^ (WST−8)	12	0.28	2.71

Note: ^a^ In this work by MTT assay after 3 h and 24 h incubation times; and ^b^ Reference [21] by WST-8 assay at 12 h incubation time.

## Supplementary Materials

The following are available online at http://www.mdpi.com/1422-0067/19/6/1596/s1.
