# Supplementary material for: Tumor Size-Dependent Anticancer Efficacy of Chlorin Derivatives for Photodynamic Therapy
Source: Int J Mol Sci. 2018 May 29;19(6):1596. doi: 10.3390/ijms19061596 (PMC6032057; doi:10.3390/ijms19061596)
Supplement: Supplementary file 1 [file ijms-19-01596-s001.pdf]

Supporting Information

Tumor Size-Dependent Anticancer Efficacy of Chlorin Derivatives for Photodynamic Therapy

Ji-Eun Chang [a,†], Yang Liu [c,†], Tae Heon Lee [c], Woo Kyoung Lee [c], Il Yoon [c,*], Kwhanmien Kim [a,b,*]

[a] Department of Thoracic and Cardiovascular Surgery, Seoul National University Bundang Hospital, Seongnam-si, Gyeonggi-do, Republic of Korea

[b] Department of Thoracic and Cardiovascular Surgery, Seoul National University College of Medicine, Seoul, Republic of Korea

[c] Nano Manufacturing Institute, School of Nanoscience and Engineering, Inje University, Gimhae, Republic of Korea

* Correspondence: kmkim0070@snubh.org Tel: +82 31 787 7131; Fax: +82 31 787 4050 (K. Kim); yoonil71@inje.ac.kr; Tel: +82 55 320 3871; Fax: +82 55 321 7034 (I. Yoon)

† These two authors contributed equally to this work.

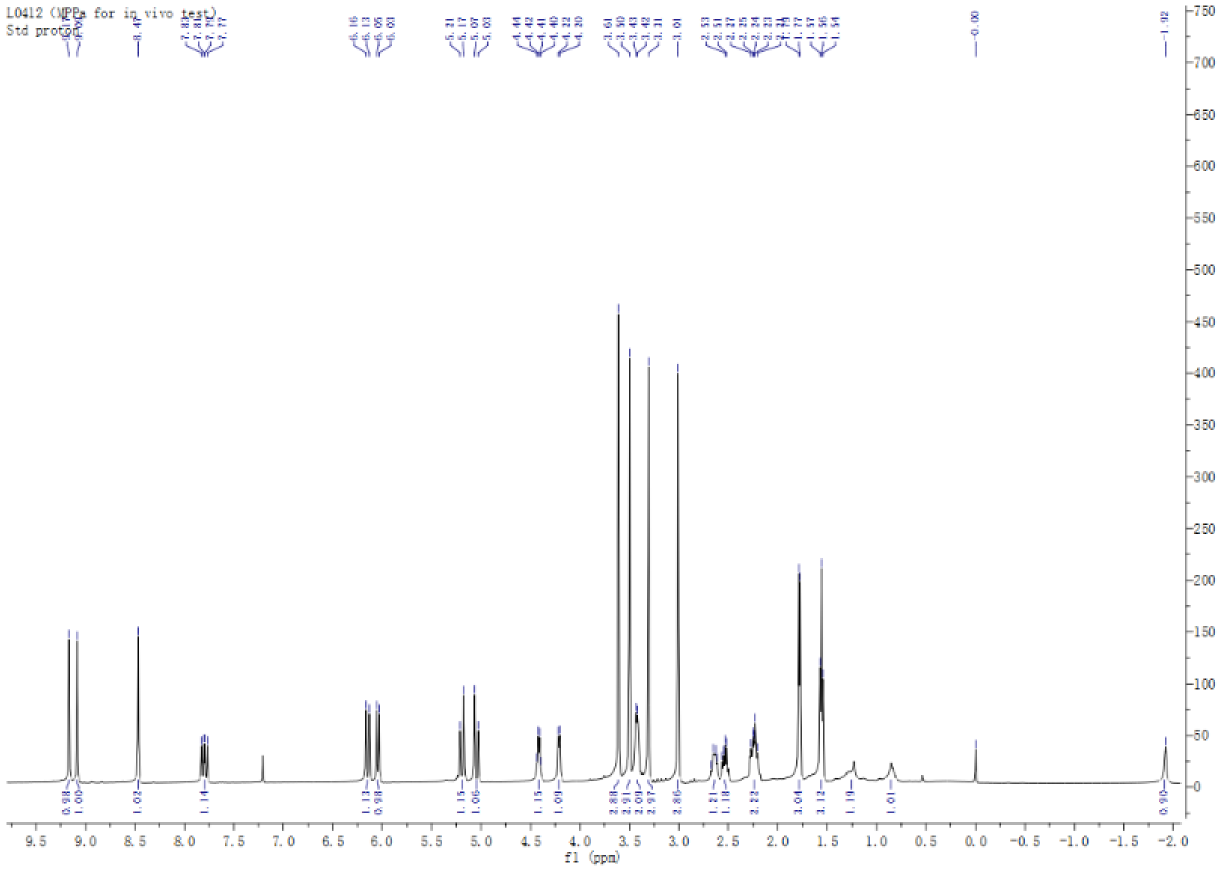

Figure S1. ^{1}H NMR spectrum of MPPa (298 K, $CDCl_3$).

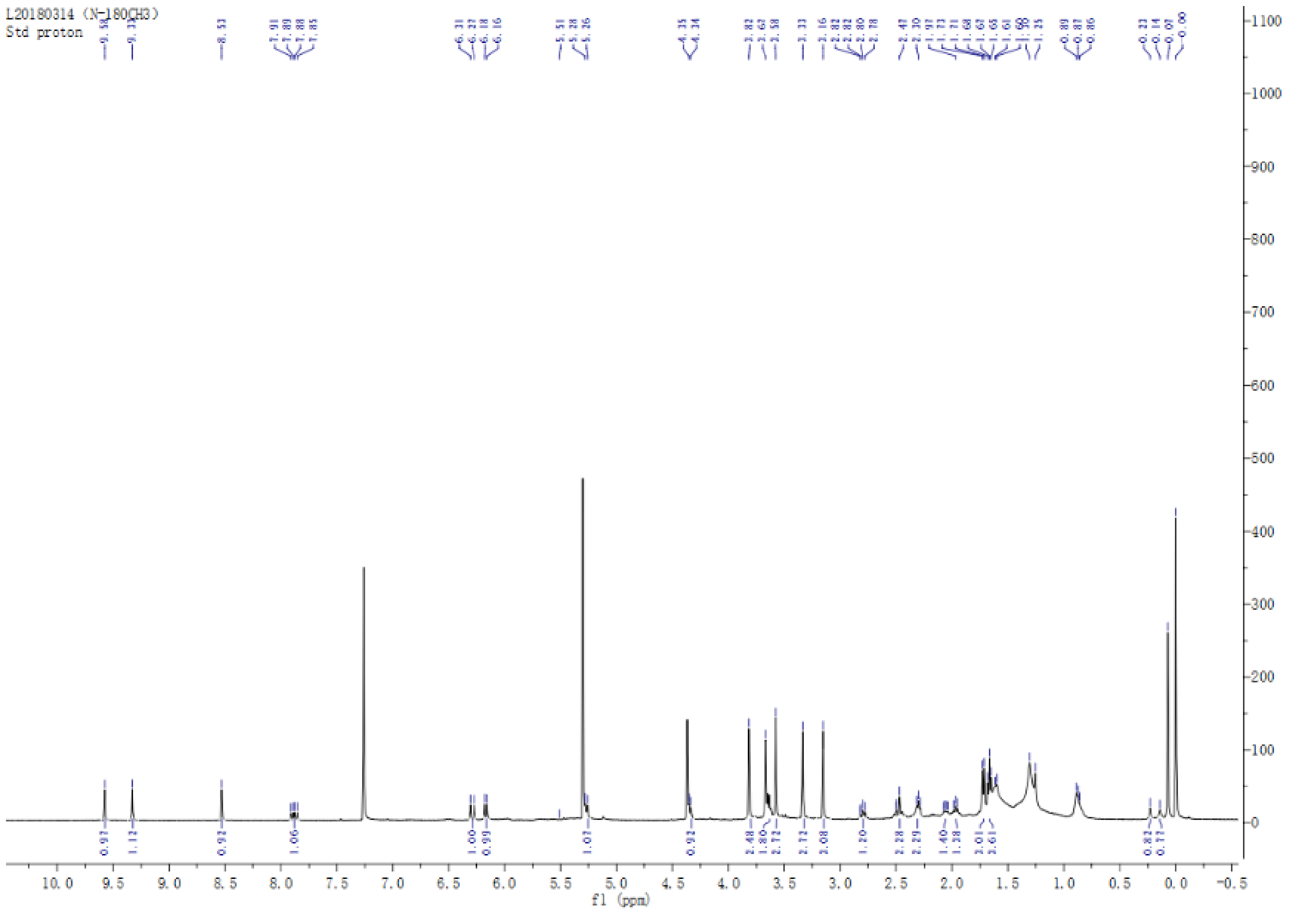

Figure S2. ^{1}H NMR spectrum of NMPi (298 K, $CDCl_3$).

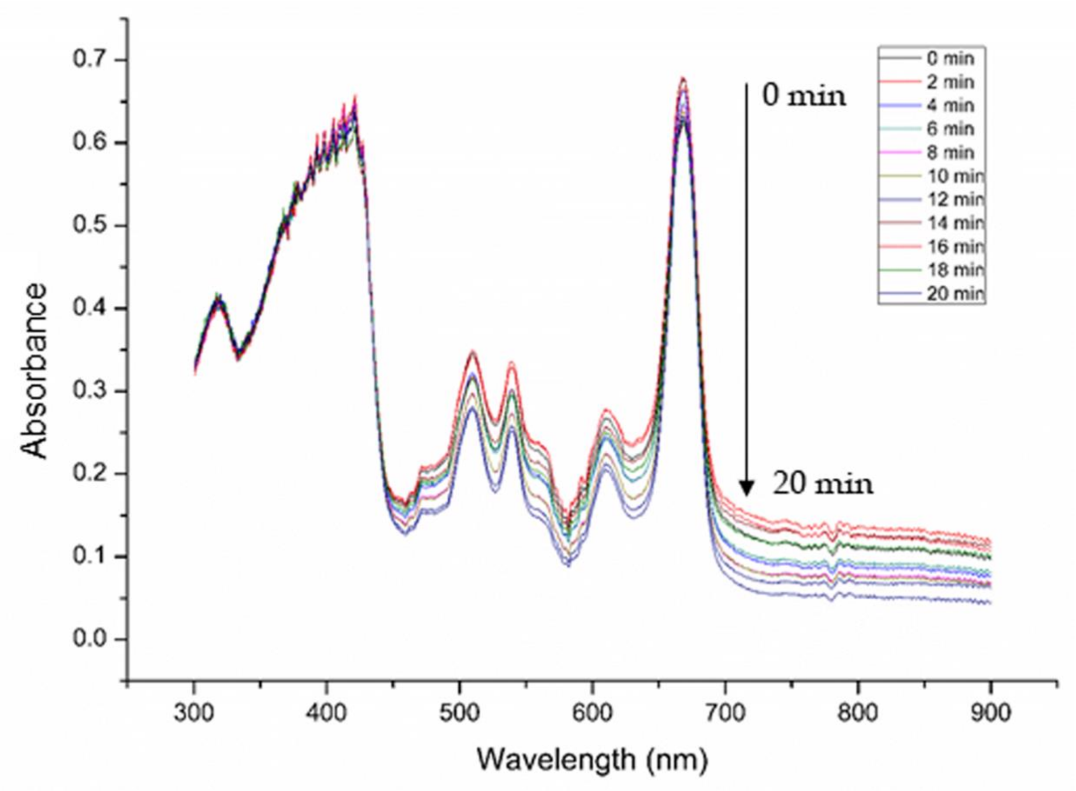

Figure S3. UV-Vis spectra for photobleaching of MPPa in CH_2Cl_2.

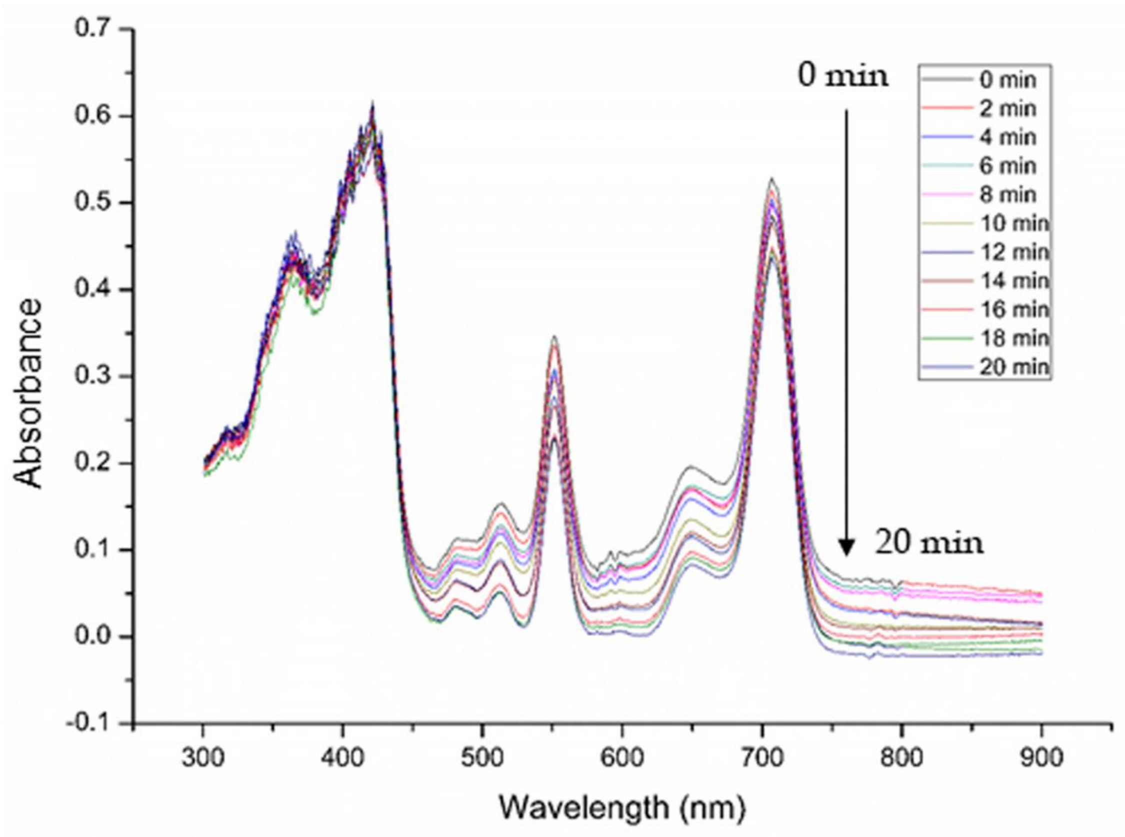

Figure S4. UV-Vis spectra for photobleaching of NMPi in CH_2Cl_2.

Table S1. Cell viability (%) of A549 cell only (control), MPPa and NMPi for photocytotoxicity against A549 cell lines at concentration range of 0.05–20.00 μM. The concentration of all compounds was 50 to 20000 nM. The percentage of cell viability was conducted by MTT assay at 3 h and 24 h incubation times after irradiation. Error values represent the standard deviation of three replicate experiments.

**Concentration (μM)**	**MPPa**		**NMPi**	
**Incubation time**	**3 h**	**24 h**	**3 h**	**24 h**
**control**	100 ± 5.77	100 ± 1.20	100 ± 5.77	100 ± 1.20
**0.05**	102.50 ± 5.55	107.59 ± 3.37	98.54 ± 4.62	102.83 ± 0.25
**0.10**	97.77 ± 4.14	103.71 ± 7.93	99.64 ± 2.00	98.21 ± 1.30
**0.25**	102.48 ± 3.39	91.01 ± 9.46	98.79 ± 3.75	102.89 ± 5.75
**0.50**	99.71 ± 4.74	31.14 ± 13.13	97.79 ± 3.37	89.02 ± 2.03
**1.00**	27.11 ± 3.53	4.47 ± 3.29	60.06 ± 6.44	13.89 ± 4.01
**2.50**	1.34 ± 1.53	1.95 ± 1.02	3.30 ± 0.60	2.07 ± 0.80
**5.00**	2.34 ± 0.72	2.46 ± 1.33	1.81 ± 1.40	1.66 ± 0.30
**10.00**	1.32 ± 1.19	1.11 ± 1.05	1.28 ± 0.78	1.40 ± 1.00
**20.00**	0.05 ± 1.27	0.16 ± 1.40	0.16 ± 0.34	0.93 ± 1.60

Table S2. Cell viability (%) of A549 cell only (control; **con**), MPPa and NMPi for dark toxicity against A549 cell lines at concentration range of 0.05–20.00 μM. The percentage of cell viability was conducted by MTT assay at 3 h and 24 h incubation times after irradiation. Error values represent the standard deviation of three replicate experiments.

**Concentration (μM)**	**MPPa**		**NMPi**	
**Incubation time**	**3 h**	**24 h**	**3 h**	**24 h**
**control**	100 ± 6.45	100 ± 5.05	100 ± 6.45	100 ± 5.05
**0.05**	107.98 ± 5.49	106.64 ± 3.68	103.19 ± 5.00	102.43 ± 3.16
**0.10**	109.29 ± 1.70	110.31 ± 7.30	107.01 ± 3.17	110.50 ± 7.85
**0.25**	110.69 ± 4.08	114.29 ± 10.26	108.73 ± 4.00	111.86 ± 2.25
**0.50**	108.93 ± 5.35	112.38 ± 7.10	109.21 ± 4.44	105.81 ± 2.40
**1.00**	108.34 ± 2.68	110.77 ± 5.33	109.53 ± 4.09	109.31 ± 6.68
**2.50**	103.45 ± 4.30	110.40 ± 3.84	109.01 ± 4.16	105.64 ± 5.92
**5.00**	100.97 ± 6.58	104.61 ± 4.20	106.04 ± 2.02	106.73 ± 1.77
**10.00**	98.20 ± 6.72	103.10 ± 5.35	104.34 ± 1.33	104.66 ± 3.91
**20.00**	86.68 ± 9.71	91.06 ± 2.90	95.31 ± 2.87	90.52 ± 7.95
